# Supplementary material for: Integrating Long-Read Structural Variant Analysis with single-nucleus RNA-seq to Elucidate Gene Expression Effects in Disease
Source: bioRxiv. 2026 Mar 23:2026.03.20.713192. Preprint. [Version 1] doi: 10.64898/2026.03.20.713192 (PMC13041997; doi:10.64898/2026.03.20.713192)

Extended Data Fig. 1

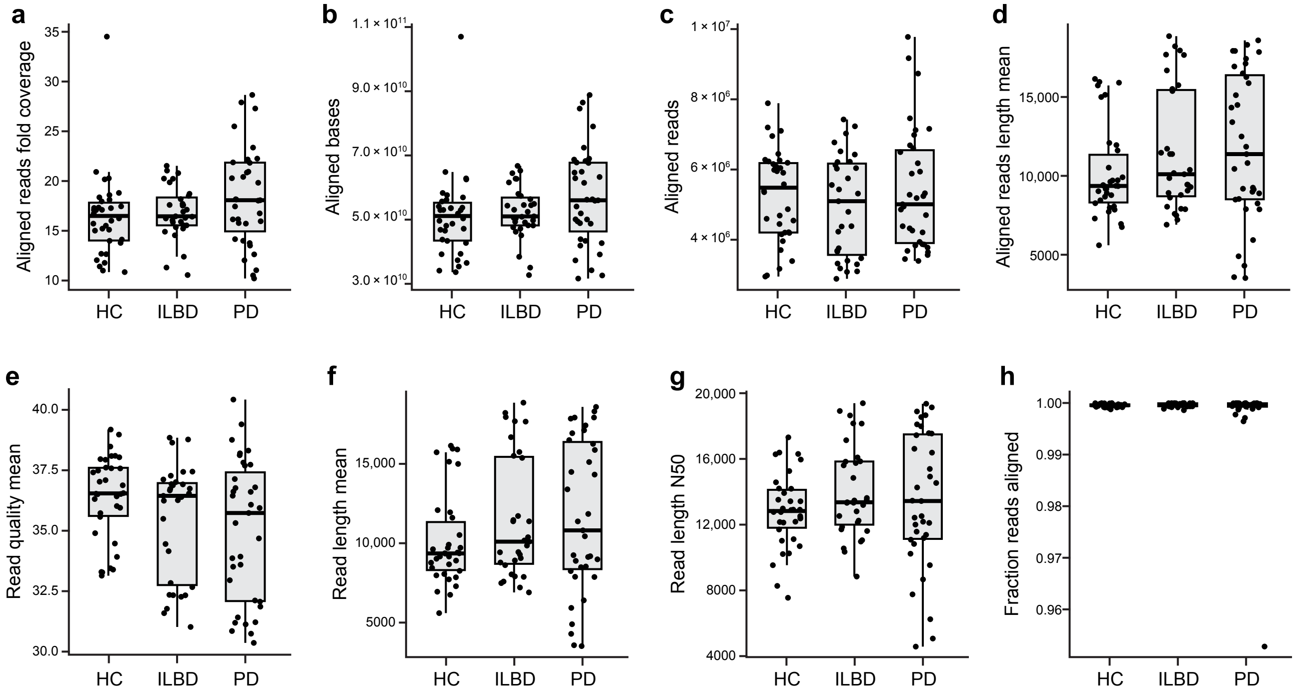

Extended Data Fig. 2

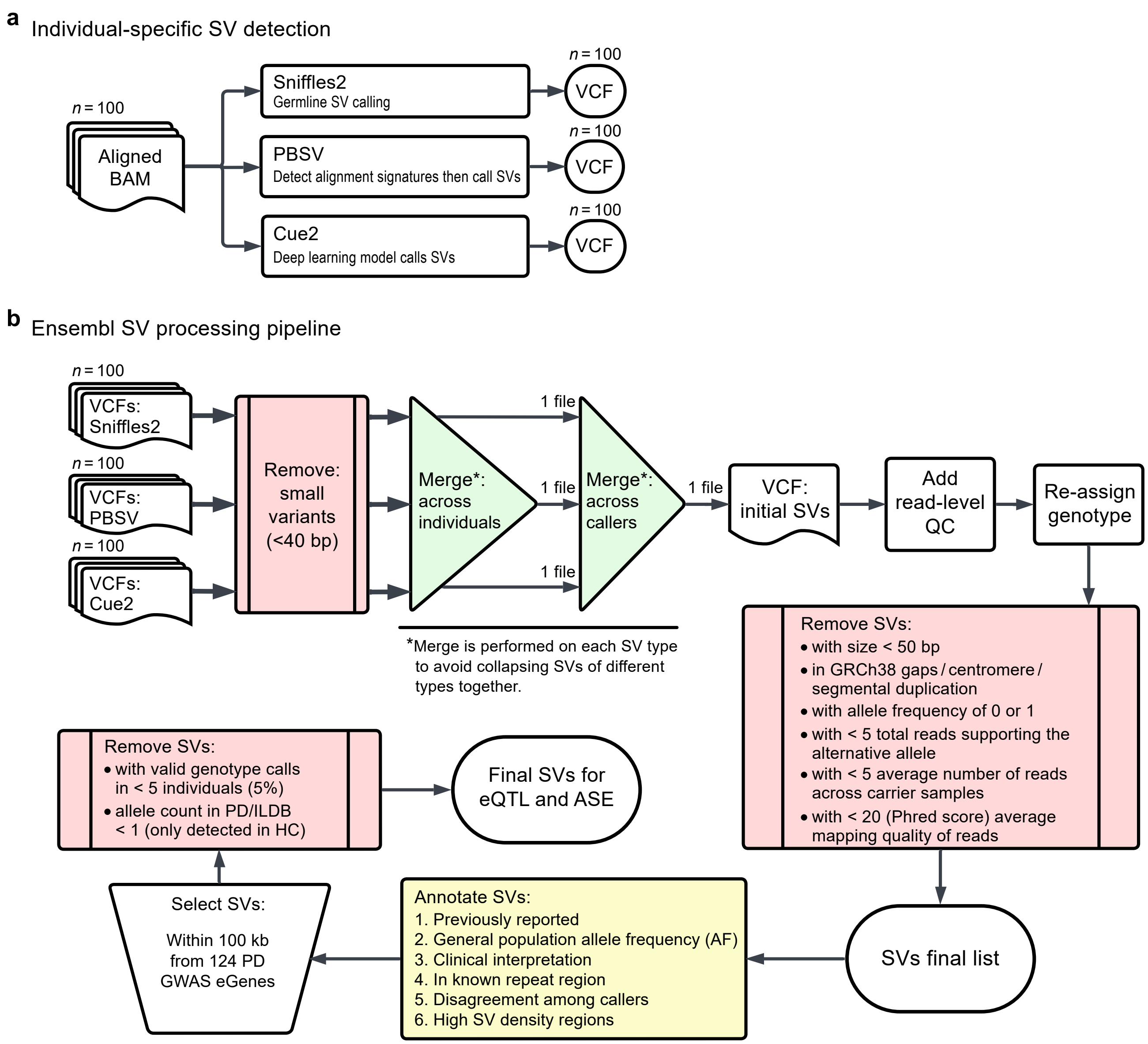

Extended Data Fig. 3

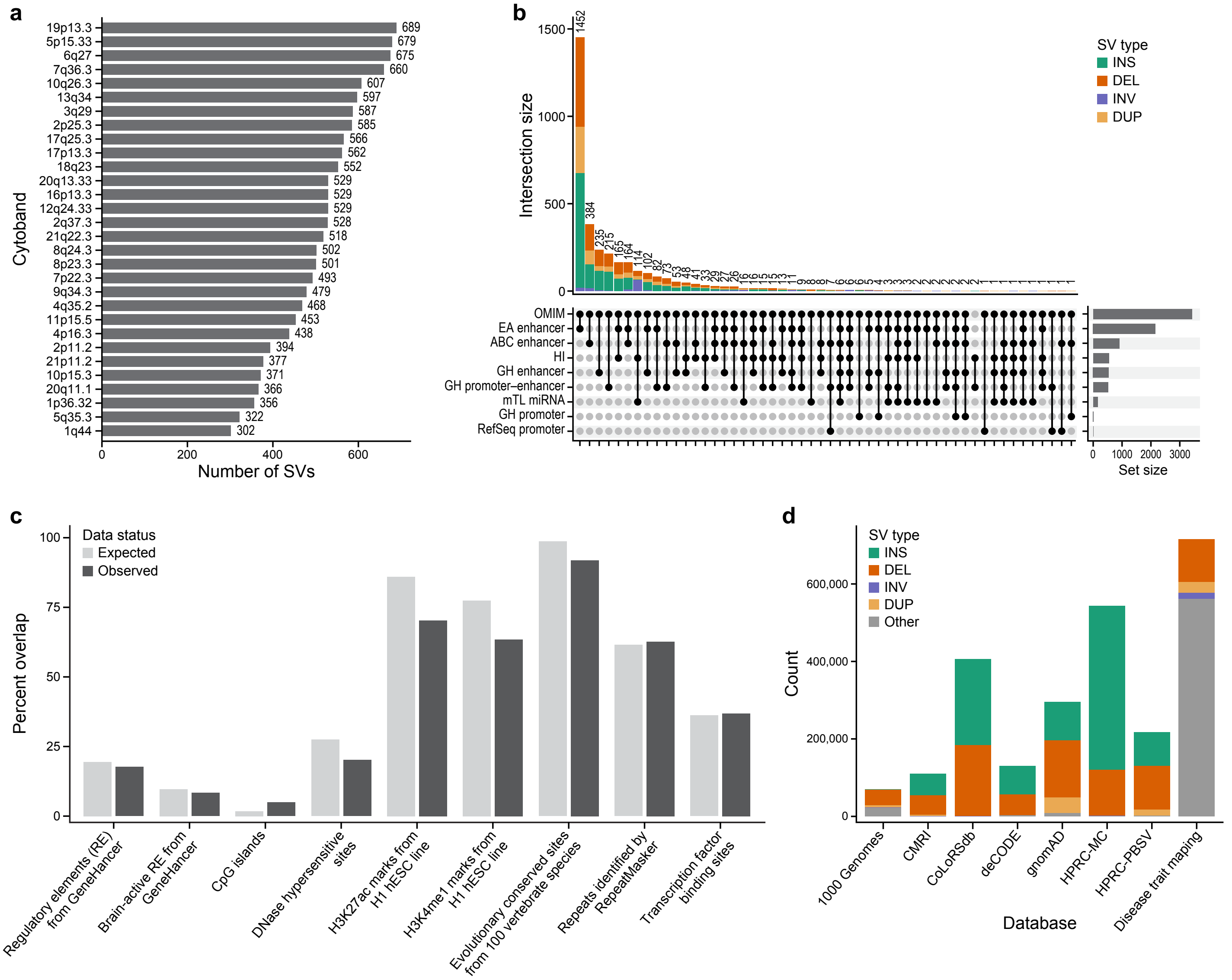

Extended Data Fig. 4

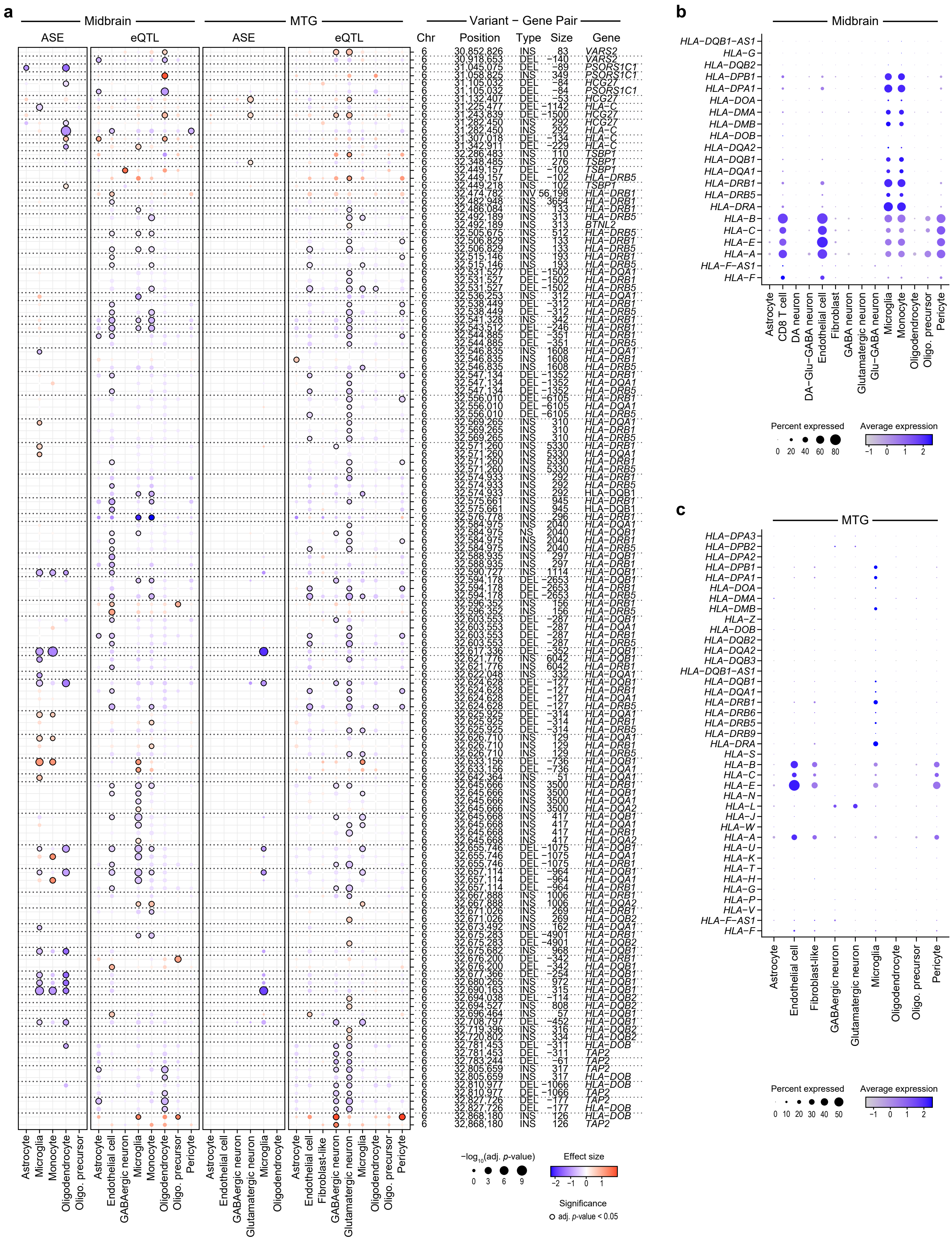

Extended Data Fig. 5

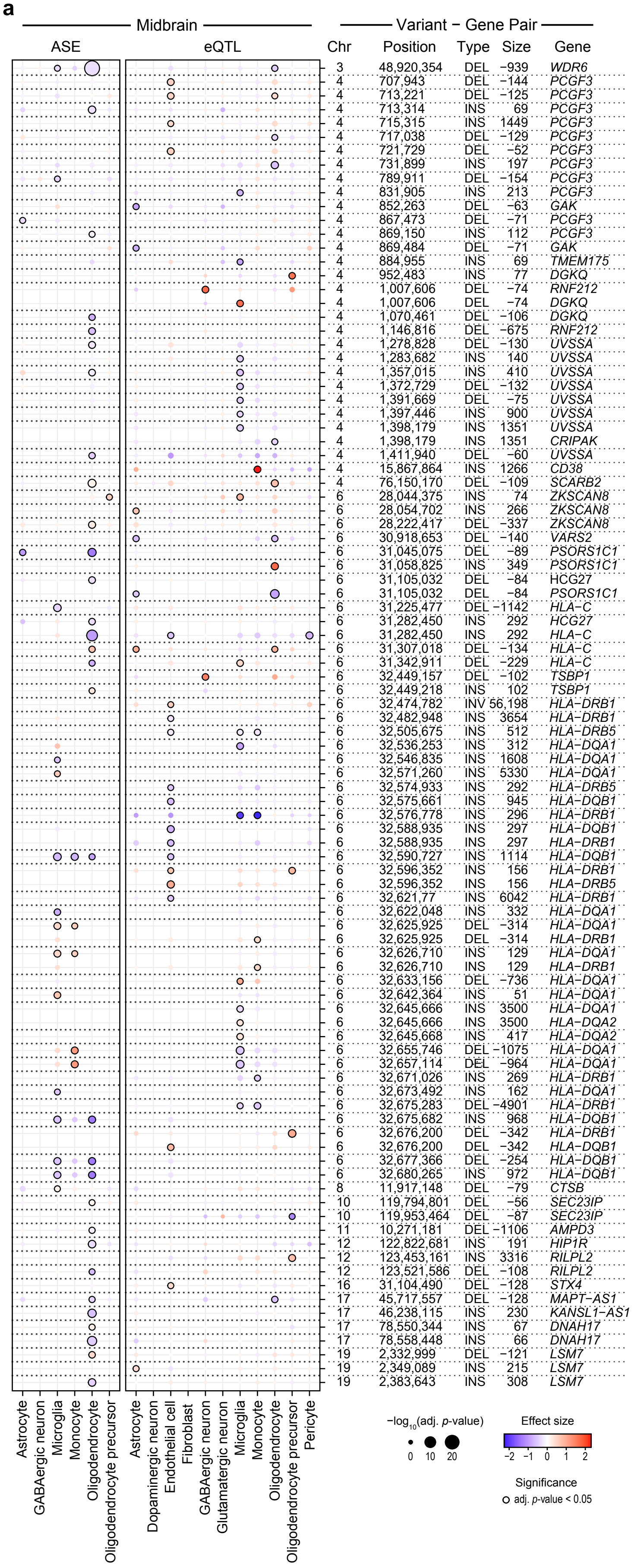

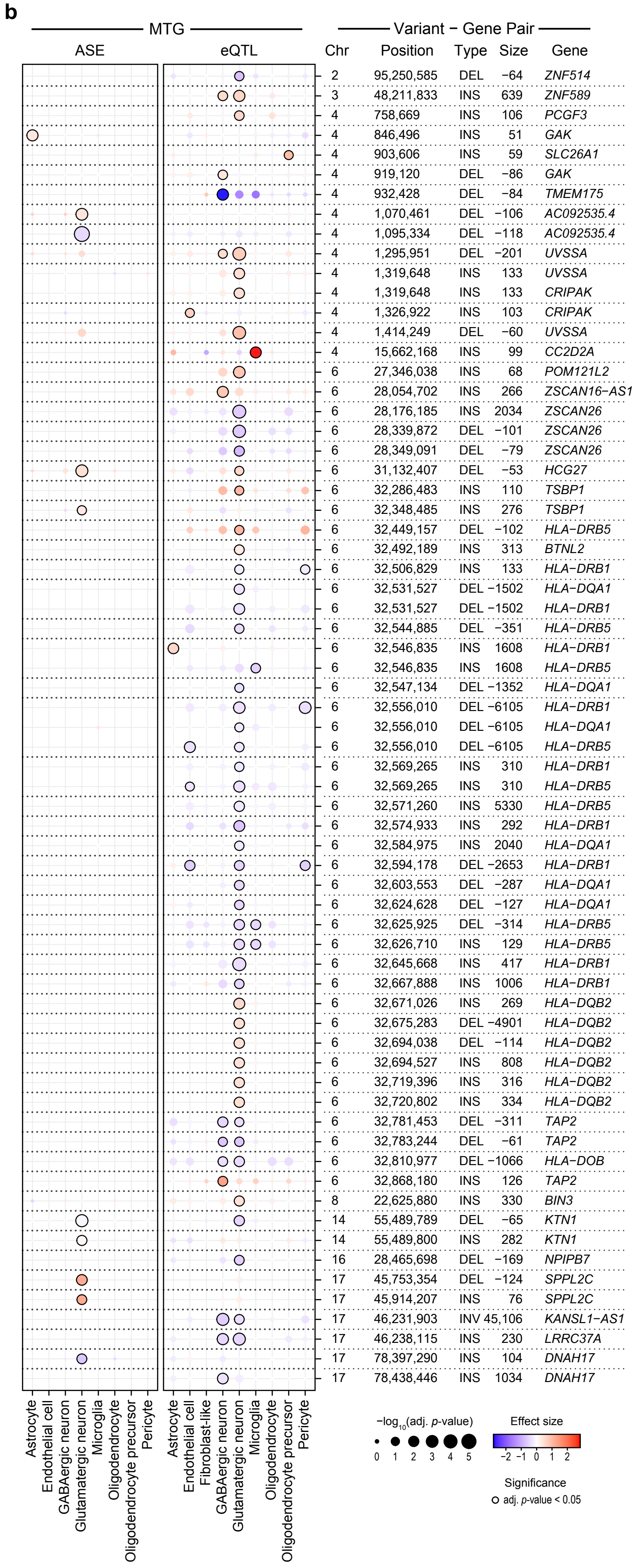

Extended Data Fig. 6

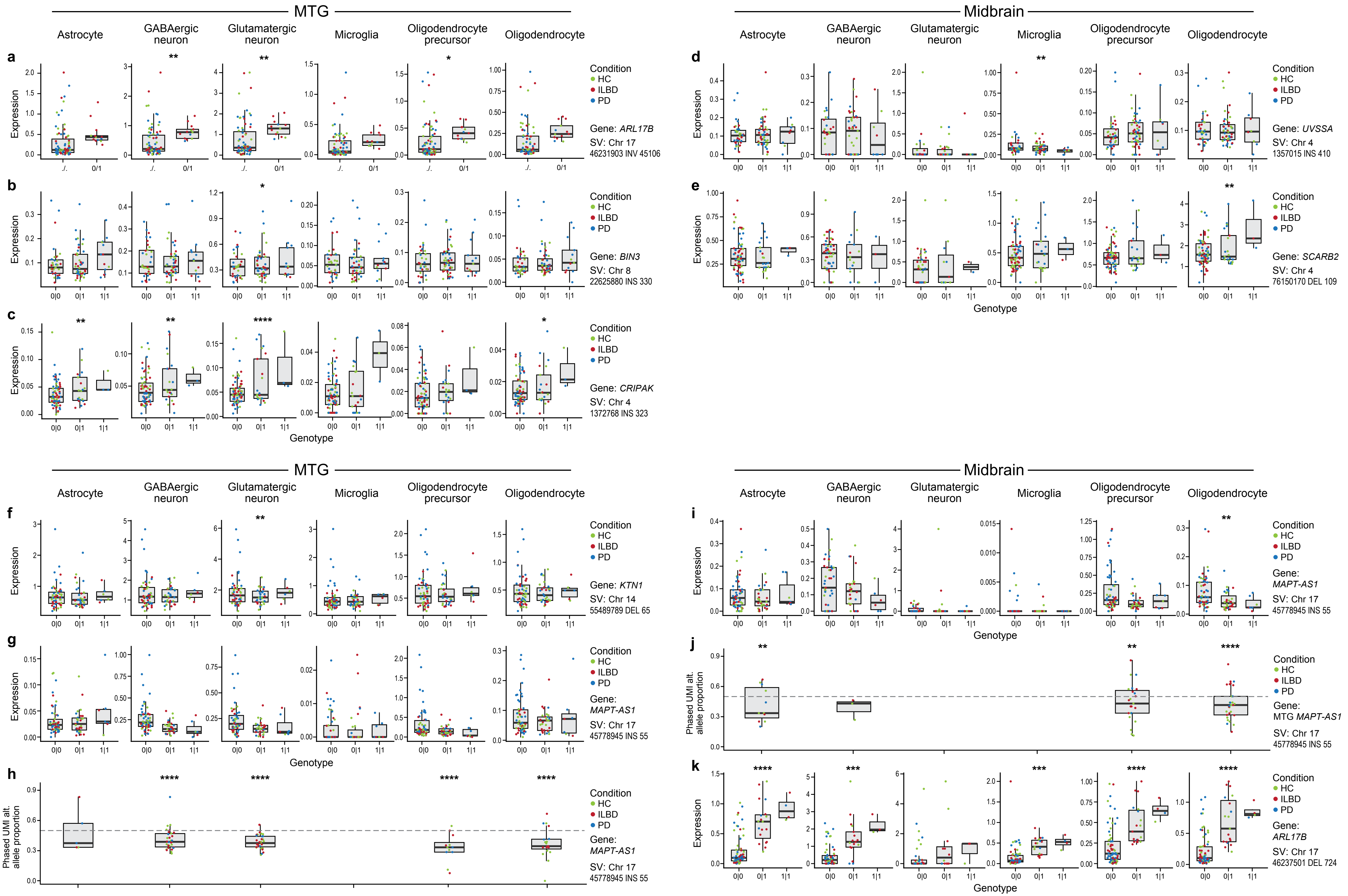

Extended Data Fig. 7

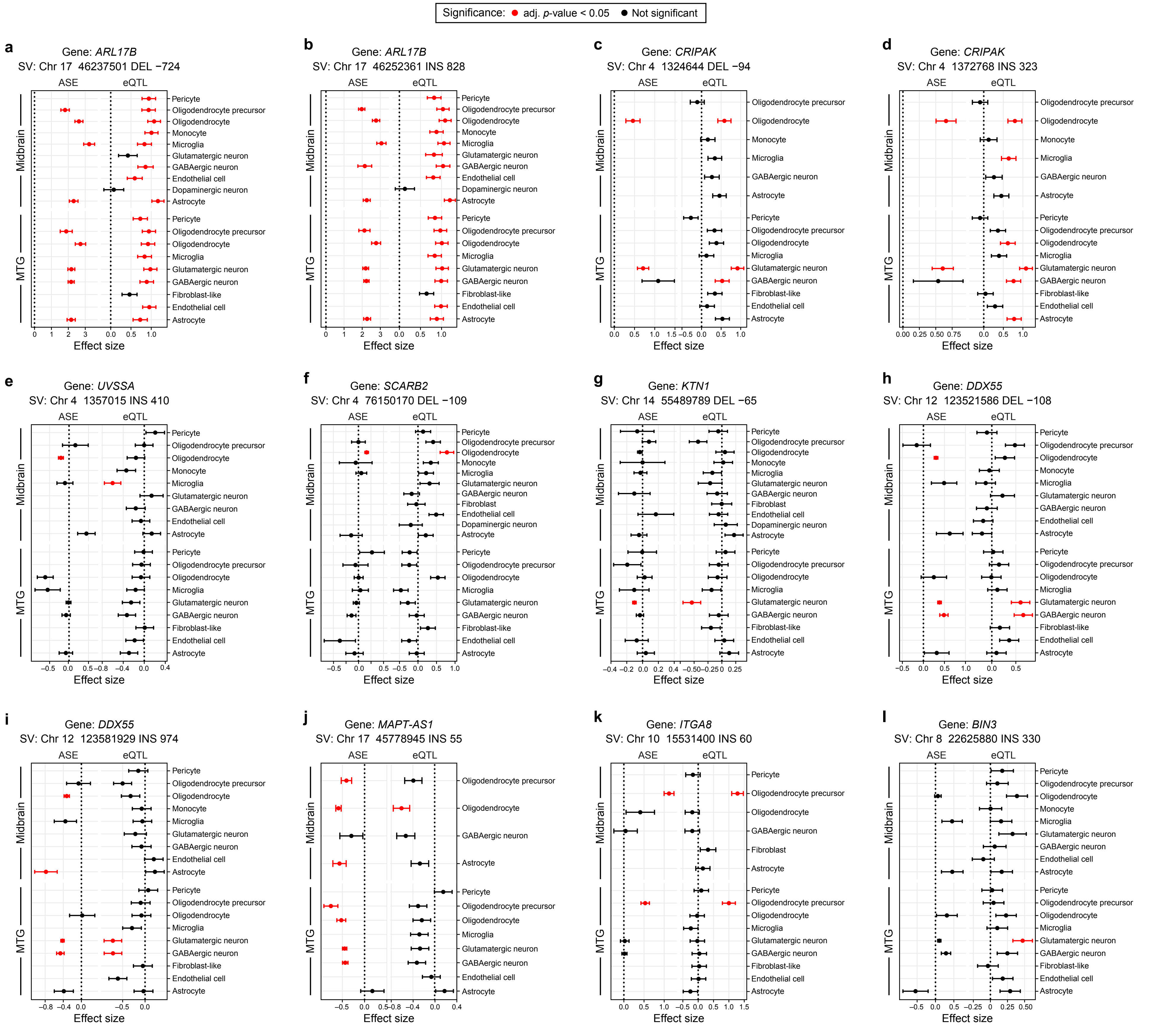

Extended Data Fig. 8

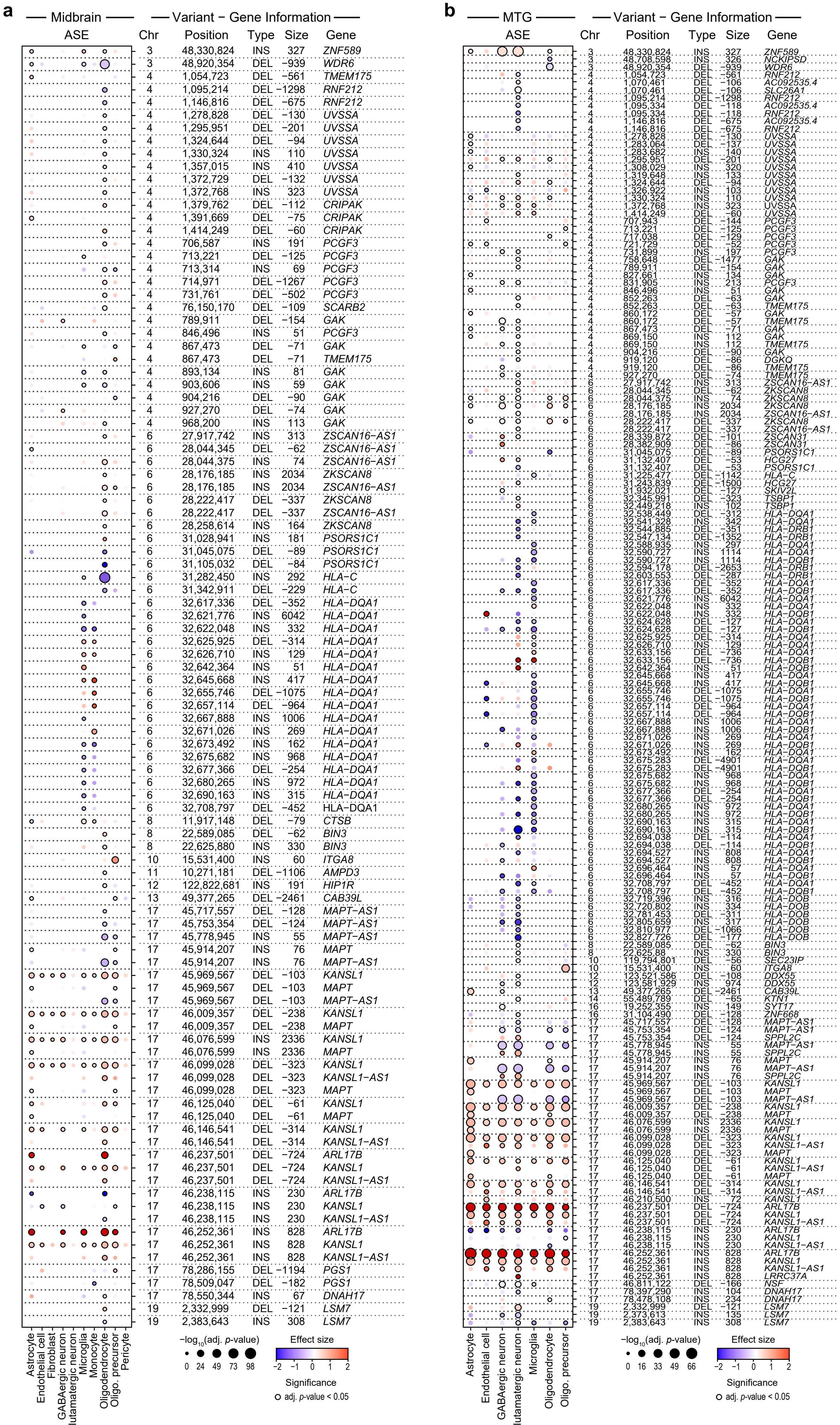

Extended Data Fig. 9

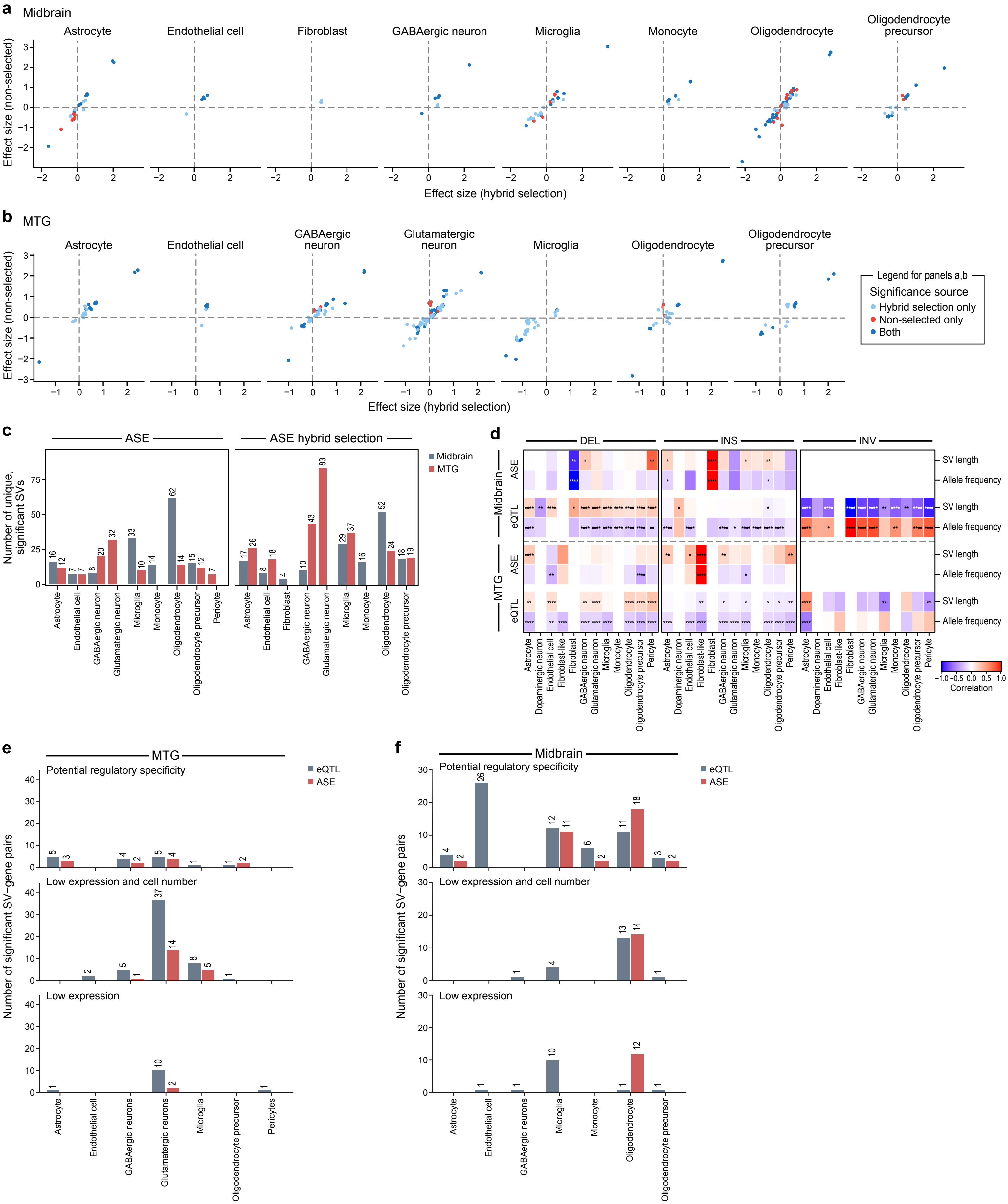

Extended Data Fig. 10

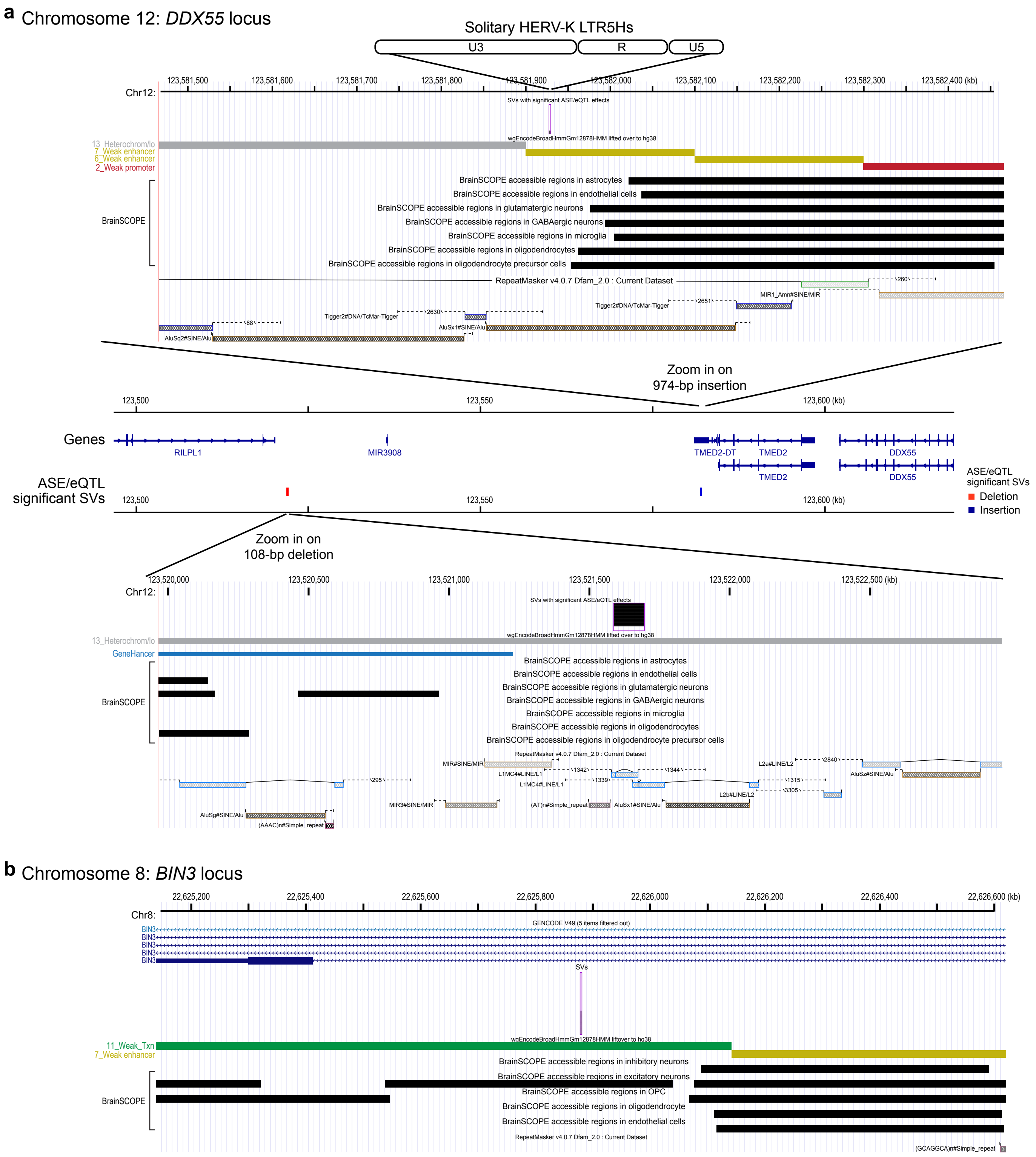

Supplement: Supplement 1 — Extended Data Fig. 1 ∣ Sequencing QC metrics. Box plots showing the quality of HiFi reads and alignment. Samples are grouped based on clinical diagnosis (x-axis). Data points show values from each sample. The metric visualized is shown by the title in each subplot with the values on the y-axis. Extended Data Fig. 2 ∣ Computational analysis pipeline. a, Graphical summary of individual-specific SV calling pipeline. b, Graphical summary of SV processing pipeline. Colors highlight key steps: SV removal (red), SV combining (green), and SV annotation (yellow). Extended Data Fig. 3 ∣ SV properties. a, Bar chart showing the top 30 cytobands with the most SVs found. The number of SVs is shown on the x-axis and the cytobands are shown on the y-axis. Exact numbers are written on the right side of the bars. b, Upset plot showing the number of SVs overlapping with regulatory elements annotated by different sources. The bubble plot lists sources of regulatory elements and shows set memberships. Black circles indicate which sets are included in a particular intersection. Upper bar chart shows the size of each intersection. Set size bar chart (right) shows the total number of SVs in each individual set. The intersection bar plot is color-coded by SV type. Source acronym: EA=Enhancer Atlas, ABC=Activity-by-contact model, HI=Haploinsufficiency, GH=GeneHancer, mTL= miRTargetLink 2.0, RefSeq=NCBI reference sequence. c, Bar chart showing the observed and expected percentage of SVs overlapping with annotated genomic features. Features include regulatory elements from GeneHancer, brain-active regulatory elements from GeneHancer, CpG islands, DNase hypersensitive sites, H3K27ac marks from the H1 hESC line, H3K4me1 marks from the H1 hESC line, evolutionarily conserved sites from 100 vertebrate species, repeat regions identified by RepeatMasker, and transcription factor binding sites. X-axis shows the genomic feature. Y-axis shows the percentage of SVs. Top 30 TFs that bind to these SV overl [file media-1.pdf]
