# Supplementary figures and images for: Integrating Long-Read Structural Variant Analysis with single-nucleus RNA-seq to Elucidate Gene Expression Effects in Disease
Source: bioRxiv. 2026 Mar 23:2026.03.20.713192. Preprint. [Version 1] doi: 10.64898/2026.03.20.713192 (PMC13041997; doi:10.64898/2026.03.20.713192)

# Supplemental Fig. 1

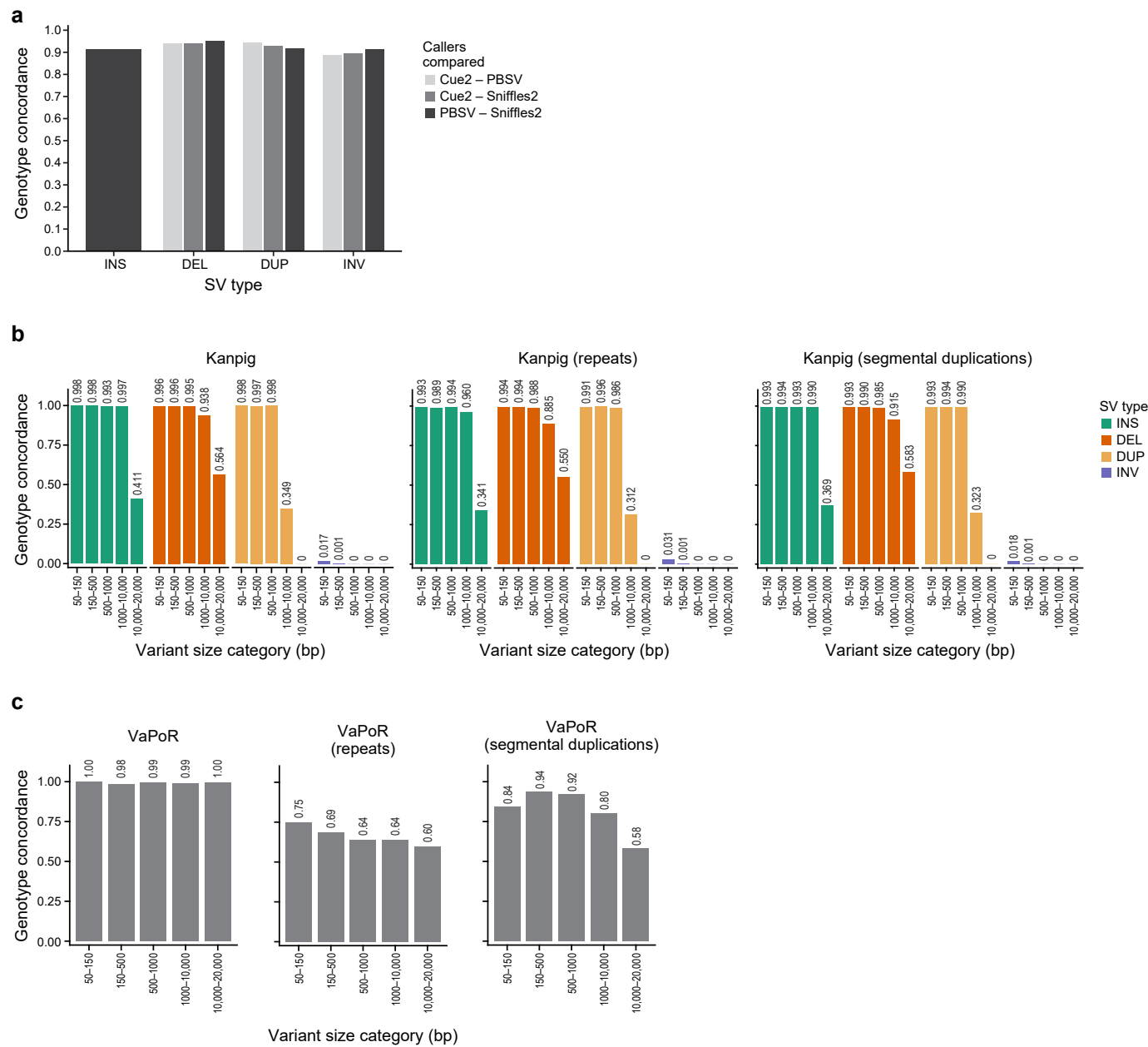

# Supplemental Fig. 2

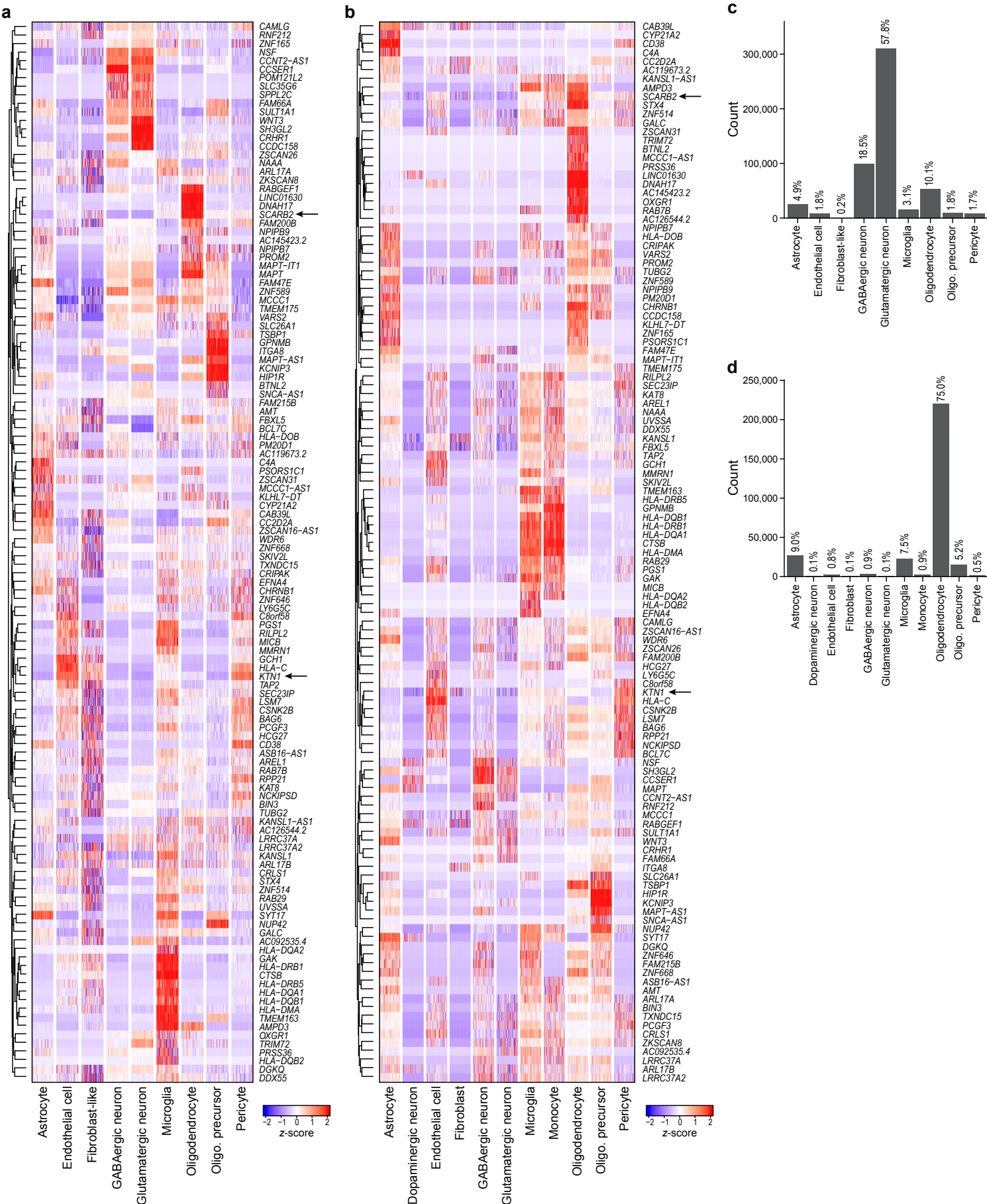

Supplement: Supplement 2 — Supplementary Fig. 1 ∣ SV caller performance. a, Bar chart showing the genotype agreement between two SV caller combinations across SV types. Different caller combinations are color-coded. SV types are shown on the x-axis. Percent of genotype agreement is shown on the y-axis. Note only one caller combination is shown for the insertions because Cue2 does not call insertions by design. b, Bar charts showing Kanpig benchmarking for synthetic datasets. Synthetic datasets include three different sequence repeat contexts: no repeat (left), short repeat (middle), and segmental duplication (right). Simulated SVs are further categorized by SV type (INS, DEL, DUP, and INV) and size (bp, x-axis). Genotype accuracy based on the ground truth is shown on the y-axis. SV types are color-coded. c, Bar charts showing VaPoR benchmarking for synthetic inversions, as in b. Supplementary Fig. 2 ∣ Cell type composition and eGene expression in snRNA-seq data. a,b, Heatmaps showing the snRNA-seq expression of eGenes across cell types in MTG, a, and midbrain, b. Rows show eGenes, shown with hierarchical clustering using Euclidean distance, and columns show cell types. Expression is scaled for each gene and z-score is shown. c,d, Bar charts showing the composition of cell types in MTG, c, and midbrain, d. Cell types are color-coded and specified on the x-axis. Y-axis shows number of cells. [file media-2.pdf]
